# Learning complex dependency structure of gene regulatory networks from high dimensional microarray data with Gaussian Bayesian networks

**DOI:** 10.1038/s41598-022-21957-z

**Published:** 2022-11-04

**Authors:** Catharina E. Graafland, José M. Gutiérrez

**Affiliations:** Instituto de Física de Cantabria, CSIC-Universidad de Cantabria, Avenida de Los Castros, 39005 Santander, Spain

**Keywords:** Gene regulatory networks, Machine learning, Probabilistic data networks, Stochastic networks

## Abstract

Reconstruction of Gene Regulatory Networks (GRNs) of gene expression data with Probabilistic Network Models (PNMs) is an open problem. Gene expression datasets consist of thousand of genes with relatively small sample sizes (i.e. are large-*p*-small-*n*). Moreover, dependencies of various orders coexist in the datasets. On the one hand transcription factor encoding genes act like hubs and regulate target genes, on the other hand target genes show local dependencies. In the field of Undirected Network Models (UNMs)—a subclass of PNMs—the Glasso algorithm has been proposed to deal with high dimensional microarray datasets forcing sparsity. To overcome the problem of the complex structure of interactions, modifications of the default Glasso algorithm have been developed that integrate the expected dependency structure in the UNMs beforehand. In this work we advocate the use of a simple score-based Hill Climbing algorithm (HC) that learns Gaussian Bayesian networks leaning on directed acyclic graphs. We compare HC with Glasso and variants in the UNM framework based on their capability to reconstruct GRNs from microarray data from the benchmarking synthetic dataset from the DREAM5 challenge and from real-world data from the *Escherichia coli* genome. We conclude that dependencies in complex data are learned best by the HC algorithm, presenting them most accurately and efficiently, simultaneously modelling strong local and weaker but significant global connections coexisting in the gene expression dataset. The HC algorithm adapts intrinsically to the complex dependency structure of the dataset, without forcing a specific structure in advance.

## Introduction

The reconstruction of Gene Regulatory Networks (GRNs) of gene expression data is an open problem and has attracted great deal of interest for decades. GRNs model interaction structure of genes in a network and are important to understand the biological functions in living organisms as well as the regulation of diseases in them. The increasing availability and improved systematic storage of gene expression data obtained with DNA microarrays^1^ revolutionized the incorporation of mathematical and computational models to model GRNs in the past two decades^2–4^. Mathematical models range from more complex models relying on sets of differential equations that directly describe dynamic changes in GRNs^5,6^ to simpler models that describe GRNs building on a graph presentation (graphical models or network models). The latter attract attention due to their capacity of visualization of the complex interaction structure of microarray data in a graph. The most simple and widely used example of a graphical model are pairwise correlation networks^7^, but lately more advanced Probabilistic Network Models (PNMs) that make use of machine learning algorithms to comprehensively model conditional and/or partial dependencies have gained in popularity.

One of the first applications of PNMs to reconstruct GRNs was done two decades ago in a seminal work^8^ using Bayesian Networks (BNs)—a subclass of PNMs relying on directed acyclic graphs— to analyze *S. cerevisiae* cell-cycle measurements using two approaches: multinomial BNs and Gaussian BNs. The former relies on discrete data and therefore requires the discretization of continuous microarray data; the latter can directly handle continuous microarray data assuming a multivariate Gaussian distribution function. However, the subsequent use of BNs in this field has mainly focused on adapting and improving algorithms for multinomial BNs^8–12^, while the application of Gaussian BNs have been left untreated (with some exceptions in a comparison study^13^). The Gaussian case though has been widely investigated in the subclass of PNMs that relies on undirected graphs, i.e., Undirected probabilistic Network Models (UNMs, also known as undirected Markov random fields or pairwise Markov networks)^14^. The most outstanding algorithm of the past decade to learn Gaussian UNMs was first applied on cell-signaling data from proteomics^15^. This algorithm is called the Graphical lasso (Glasso) and estimates the inverse covariance matrix, also concentration or precision matrix, of the Gaussian distribution function. It was successfully applied in the high dimensional setting of gene interaction networks^16,17^ and in network clustering in bioinformatics^18^, but also in psychology networks^19^, risk management^20,21^, and climate^22^.

The application of Gaussian UNMs to continuous microarray data has faced two main challenges. First, the large-*p*-small-*n* nature of the data. Microarray experiments contain expression levels of thousands of genes at the same time, but have relative small sample size. Glasso deals with high dimensional data by imposing sparsity on the precision matrix and thus on the undirected graph. Secondly, degrees of interaction among genes in gene networks are not uniform, but higher order. In GRNs, Transcription Factor encoding genes (TFs) regulate the expression of many target genes. However, only a few genes encode TFs, which can be seen as gene hubs^23,24^ that confer GRNs with scale-free network characteristics, as suggested by previous research on the degree distribution of GRNs^25^. Numerous modifications of the Glasso algorithm distribution have been proposed in recent years to better model this complex structure of interactions in microarray data (and other real-world datasets). Two of them were developed especially for gene regulatory networks that are expected to be scale-free^26^ or to consist of hubs^27^. We will refer to them as the Scale-Free Glasso (SFGlasso) and the Hub Glasso (HGlasso). Both modifications of the Glasso algorithm force beforehand the expected structure in the estimation of the precision matrix and were shown to outperform Glasso on simulated data that had these characteristics.

As an alternative, in this paper we build on the initial work using Bayesian Networks^8^ and propose the use of Gaussian Bayesian Networks (GBNs) to address the complex structure problem in GRNs. Our motivation stems from a recent paper showing the suitability of GBNs for modelling the complex (hub) interaction structure in complex high dimensional data, revealing the underlying probabilistic backbone^28^. Here we use the score-based Hill Climbing (HC) GBN learning algorithm used in that work, which shows good performance when compared with other GBN learning algorithms^29^, and assumes no specific structure beforehand. We compare HC with the default approach for learning sparse UNMs, the Graphical lasso (Glasso) and with its modifications SFGlasso and HGlasso using two different datasets. On the one hand, we use simulated data from an in silico gene regulatory network allowing for a comprehensive evaluation of the algorithm. On the other hand, we use (the most-up-to date version of) the dataset^30^ from the *Escherichia coli* (*E. coli*) genome, which is entitled to be the best known/encoded organism on earth and from which information on TFs is well documented.

This simultaneous study and intercomparison of direct and undirect structure learning algorithms for high dimensional complex data is, to our knowledge, new. First, we place the algorithms in the broader perspective of PNMs and analyse their learning method, the statistical criterion and form of presentation (directed or undirected network). We then evaluate the algorithms in terms of accuracy and data explanation capabilities, paying attention to the compactness and sparsity of the learned networks and to the truthful balancing of dependencies of different orders.

## Results

### BNs and PNs in the general framework of Gaussian probabilistic network models

**Figure 1:**
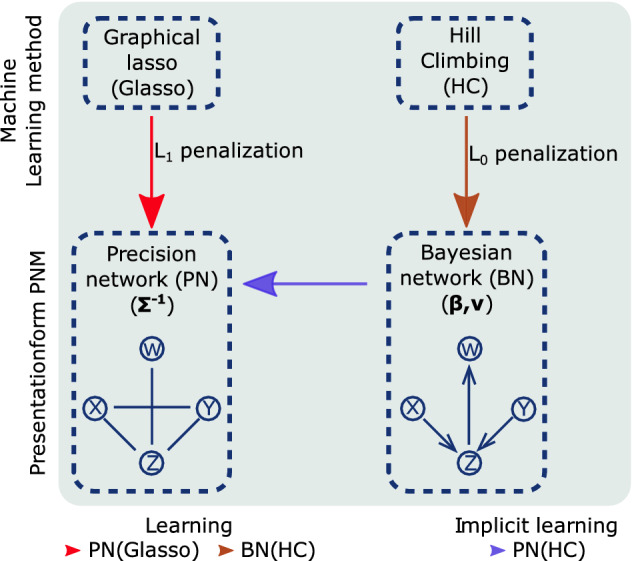
Schematic illustration of the presentationforms of Gaussian probability density function ${\text {P}}$ in terms of conditional parameters $({\varvec{\beta }},{\varvec{\nu }})$ and precision matrix $({\varvec{\Sigma }}^{-1})$, associated with Bayesian Networks (BNs) and Precision Networks (PNs), respectively. Brown and red arrows refer to the associated learning algorithms (brown; score-based Hill Climbing algorithm, red; Graphical lasso) together with the type of integrated statistical criterion ($l_0$ or $l_1$ penalization). The purple arrow refers to the analytical transformation of the parameterset from BNs (learned with Hill Climbing) to PNs.

Gaussian probabilistic network models^31^ consist of graph and parameterset. The parameterset fully determines the associated Gaussian Joint Probability Density (JPD) function. The type of parameterset determines the presentationform of the graphical model, i.e. the ‘meaning’ of the edges in the graph. A Gaussian JPD function can be determined by different types of parametersets such as the covariance matrix $({\varvec{\Sigma }})$, its inverse, the precision or concentration matrix $({\varvec{\Sigma }}^{-1})$, or linear regression coefficients in combination with local variation coefficients $({\varvec{\beta }},{\varvec{\nu }})$. Figure 1 shows two learning methods and their associated presentationforms of the Gaussian JPD function: Precision Networks or pairwise Markov networks (PN $({\varvec{\Sigma }}^{-1})$) and Bayesian Networks (BN $({\varvec{\beta }},{\varvec{\nu }})$). In this study we leave out the analysis of correlation networks that rely on the $({\varvec{\Sigma }})$ presentation generally generated from a thresholded sample correlation matrix, because they cannot properly regulate high dimensional data. We refer the interested reader to Graafland et al.^28^ for an extensive comparison study between BNs and correlation networks. The representations of the Gaussian JPD function in terms of $({\varvec{\Sigma }}^{-1})$ and $({\varvec{\beta }},{\varvec{\nu }})$ are described in “Methods” section “Gaussian probabilistic network models” and subsections “Probabilistic PN models” and “Probabilistic BN models”.

As illustrated in Fig. 1, the algorithms under subject of study, Glasso (and modifications) and Hill climbing, differ in the parameterset (and hence the presentationform) they have as an objective to learn. They do however coincide on their machine learning spirit; both algorithms attempt to find a parameterset that optimizes a score function in which a statistical criterion (penalization) is integrated. The score functions and their statistical criteria are described in more detail in “Methods” sections “Learning PN structure from data” and “Learning BN structure from data”. Learning methods (the steps in the algorithms) are chosen to efficiently execute this optimization task and are described in “Methods” section “Learning with hill climbing and glasso”. In the case of Hill Climbing the search space is restricted to that of Directed Acyclic Graphs (DAGs) and in the case of Glasso to that of symmetric positive definite precision matrices, which, converted to binary format, are translated into undirected graphs. These constraints procure that the edges in the networks respectively are associated with the regression coefficients ${\varvec{\beta }}$ in the $({\varvec{\beta }},{\varvec{\nu }})$ parametrization of the JPD function and with the entries of the precision matrix ${\varvec{\Sigma }}^{-1}$ in the $({\varvec{\Sigma }}^{-1})$ parametrization.

Once learned, a PNM is in general not bound to its initial parametrization/presentationform. At some cost, an analytical transformation of the parameterset can transform the presentationform of the PNM. In Fig. 1 the purple arrow represents a direct transformation between parametersets from BNs to PNs for which an analytical formula exists. The analytical transformation is described in “Methods” section “Transformation of probabilistic BN model to probabilistic PN model”. The transformation makes the initial BN loosing some information on its independency structure (see how the graphs of BNs and PNs encode independence statements about the JPD function in “Methods” section “Dependencies in BN and PN structure”), but allows us to compare Hill Climbing and Graphical lasso under the same UNM framework.

### Data, reference networks and algorithm settings

In this study we use two gene expression datasets, a synthetic benchmark dataset and a real-world dataset. The former is a gene expression dataset generated from an in silico network provided in one of the DREAM (Dialogue on Reverse Engineering Assessment and Methods) challenges (https://dreamchallenges.org). DREAM is a community-driven initiative to promote open, reproducible, and collaborative scientific research in biomedicine, supplying challenges that address a broad set of questions in healthcare and the life sciences. A significant part of the challenges is oriented to train machine learning models that could predict gene expression from sequences. The in silico network in the DREAM5 challenge consists of a total amount of 1548 genes of which 178 genes encode TFs and count as the origin of all transcriptional regulation interactions. Since all interactions go from TF encoding genes to target genes, the in silico network is by nature a directed network consisting of 4012 edges (unique interactions) without self-loops. This gold standard was used to generate a simulated gene expression dataset (with sample size $n = 805$) using the Gene Net Weaver^32^, including random expression levels for 95 additional genes (17 decoy TFs and 78 decoy target genes).

The real-world dataset is constructed from two public available datasets for the *Escherichia coli* (*E. coli*) genome. On the one hand we use the *E. coli* microarray dataset that is available from the Many Microbe Microarrays database (M3D)^1^. This dataset contains the uniformly normalized expression levels of 4297 genes in *E. coli* measured under 466 experimental conditions using Affymetrix arrays. Expression levels from the same experimental conditions are averaged and their mean expression provides one of the 466 sample points in the dataset. In particular we use the latest version at this moment: E_coli_v4_Build_6. On the other hand, a reference network to evaluate the learned PNMs is constructed from a dataset containing evidence about transcriptional regulations (i.e. interactions that arise from TF encoding genes and their target genes). For the *E. coli* genome, a number of studies generated information on transcriptional regulations. The Regulon Data Base (RegulonDB) is the primary database^33^ in which this information is gathered. We use the most complete file in the RegulonDB called network_tf_gene.txt.

We quire the algorithms to construct networks using only the expression levels in M3D of the 1683 genes from which evidence is reported in the RegulonDB. The known interactions in the RegulonDB will then really act like a reference network that enables us to evaluate the accuracy of the learned GRNs from the microarray data. A similar strategy is applied in^30^. The RegulonDB network has similar characteristics as the in silico network in DREAM5. However, in contrast to the in silico network, the RegulonDB network is not ‘perfect’, i.e. there are ‘false negatives’ in the gold standard due to undiscovered relationships. The 1683 genes in the reduced M3D dataset contain a total of 173 TF encoding genes of which 172 count as the origin of all transcriptional regulation interactions in the reference network. Together they are good for 3381 unique interactions without self-loops. As interactions go from TF encoding genes to target genes, the reference RegulonDB network is by nature a directed network.

The number of edges that is needed to construct a PNM from the microarray dataset plays an important role in the quality and the practical possibilities of a structure learning algorithm. With this in mind we generate networks of different sizes for all algorithms. To vary the amount of edges $|\mathrm {E}|$ we vary the initial parameters $\lambda$, and $\lambda _1, \lambda _2, \lambda _3$, and $\alpha$ for Glasso, HGlasso, and SFGlasso, respectively (see “Methods” section “Learning PN structure from data”). For Hill Climbing we obtain networks of different amount of edges by varying the amount of iterations while using the standard $\mathrm {BIC}$ score (an action that give similar results as application of the $\mathrm {BIC}_{\gamma }$ score and varying the parameter $\gamma$^29^). The directed BNs learned by Hill Climbing are transformed to UNMs for evaluation. This process, described in “Methods” section “Transformation of probabilistic BN model to probabilistic PN model”, has a small penalty (extra, unnecessary parameters/edges will be added), but allows for a fair comparison with other algorithms (SF-, H- and Glasso) as the transformed edges will encode the same type of parameters.

### Results for in silico benchmark network in DREAM5

We first assess the performance of the algorithms using the amount of True Positive edges (TPs) according to the benchmark in silico network. To this aim, an undirected edge in the learned network is a TP if there exists an associated directed edge in the in silico network. Moreover, the amount of connected Transcription Factors (TFs) in the estimated network is measured as an indication of the hub structure of the learned networks. These measures are analyzed as a function of the network size $|\mathrm {E}|$ to assess the capability of the methods to produce accurate, though compact and sparse networks.

**Figure 2:**
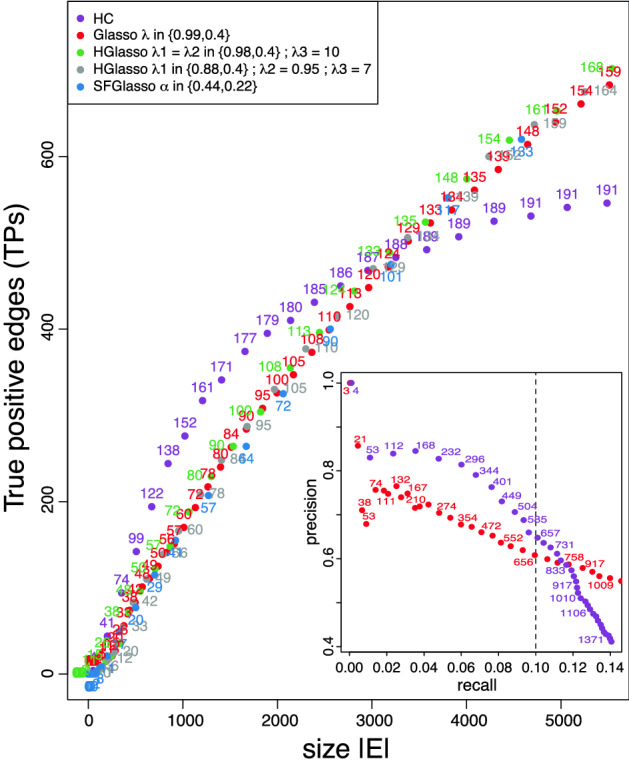
True positives (dots) and amount of transcription factors (numbers) w.r.t. the networksize $|\mathrm {E}|$ as encountered from the in silico dataset by HC (purple), Glasso with $\lambda \in \{0.98,0.6\}$ (red), HGlasso with $\lambda _1 = \lambda _2 \in \{0.98,0.6\}$, $\lambda _3 = 10$ (green), HGlasso with $\lambda _1 = 0.9$, $\lambda _2 \in \{0.61,0.55\}$, $\lambda _3 = 14$ (orange), HGlasso with $\lambda _1 \in \{0.98,0.6\}$, $\lambda _2 = 0.95$, $\lambda _3 = 7$ (grey), and SFGlasso with $\alpha \in \{0.43, 0.3\}$ (blue). The inset shows the precision vs the recall values for HC and Glasso with numbers indicating the network size; note that the the 0.1 recall value is indicated with a dashed line.

Figure 2 shows the results on TPs and TFs with respect to the number of total estimated edges $|\mathrm {E}|$ for networks learned with the four algorithms. The dots indicate the amount of TPs and the dot labels, in the form of numbers, indicate the amount of TFs found in the corresponding network. This figure shows that the UNMs obtained with HC exhibits the best results (highest amount of TPs and TFs) for sparse networks up to 3000 edges. The biggest gain of TPs with respect to the amount of edges $|\mathrm {E}|$ occurs in the range from 500 to 1500 edges. In this range also the amount of recovered TFs is highest (more than 10 new TFs per 100 added edges). This simultaneous increase of TPs and TFs is intuitive as all 4012 edges in the in silico network consist of at least one TF. The standard Glasso algorithm recovers less TP edges than HC in sparse networks, with recovering rates particularly lower than HC in the range of 500–1500 edges; this could be related to the fact that in this range Glasso only recovers between 1 and 5 new TFs per 100 edges. In bigger networks consisting of more than 3000 edges the amount of TPs exceeds HC values. Note that all Glasso variants exhibit a similar performance as the standard Glasso. However, their search strategy is clearly different: the TPs of SFGlasso are originated by less TFs than Glasso, whereas the TPs of HGlasso are originated by more TFs than Glasso.

In addition to this analysis, we also evaluate learned networks using their precision and recall. The precision of a network shows how many of the predicted TF–target-gene links (edges in the learned network) are true positives (relative to all predicted positives), whereas the recall shows the rate of TPs relative to the total amount of positives. The use of the Precision-Recall curve (PR-curve) is in concordance with the methodology used in DREAM5 (see “Methods” section “Evaluation measures” for the formal definition of these measures). To benchmark our results in the framework of the DREAM5 challenge, we eliminated all links between two target-genes from the networks’ edgelist before calculating their precision and recall. Note that participants in DREAM5 were provided with the names of the TFs in the set of genes and algorithms in DREAM5 were queried to search only for TF–target-gene relationships in the gene expression data; in this work we quire the algorithms to learn PNMs consisting of undirected edges in which, despite TF–target-gene relationships, also target-gene–target-gene dependencies are included. These edges are found in gene expression data due the existence of co-regulated genes by the same TFs^2^. The inset of Fig. 2 displays the PR-curve for the HC and Glasso algorithms. Each network in the curve is labeled with its associated amount of retrieved (predicted positives) TF–target-gene relationships. The figure shows a trade-off between precision and recall for all learned networks. For a recall below 10%, HC finds sparse networks of high precision (i.e. much qualitative information in sparse networks). On the other hand, Glasso finds networks of lower precision for a recall below 10%, but shows a steadier decay in precision for recall above 10%. Variants of Glasso behave equally to Glasso and are not displayed in the inset.

Although many GRN inference algorithms that participated in DREAM5 used (lasso) regression techniques, none of these methods are comparable to Glasso and HC algorithms used in this work, because they mainly focus on the regression of TFs on individual target genes. HC and Glasso perform in the top 15 network inference methods used in DREAM5 for recall values below 10% as judged by Fig. S3a of Supplementary Note 4^2^. This benchmarking is not perfect, as our algorithms did not receive information about the TFs in advance and are therefore at a disadvantage compared to the algorithms that participated in DREAM5.

### Results for real-world gene expression data

The evaluation results for the in silico network highlight HC as an promising exploratory algorithm (finding sparse networks of high precision for recalls below 10 per cent), whereas Glasso and variants seem to have less exploratory ability (exhibiting lower precision for sparse networks) but to perform more steady after an initial exploration phase (medium precision for networks of growing size and higher recall). In this section we evaluate the algorithms on real-world M3D data using evaluation measures chosen in alignment with the nature of real-world datasets and the focus of this work: complex gene expression datasets of low sample size without background knowledge on which we want to chart the exploratory capability of the algorithms. We do not consider false positives in this case (and thus do not consider the PR-curve) since the networks are learned from gene expression data that also contain target-gene–target-gene dependencies that might not be in conflict with the evidence in the true network due to the existence of genes co-regulated by the same TFs. As these dependencies are not reported in the true network, they would be regarded as false positives, although they might be correct. Moreover, in the case of real-world data, the algorithms may discover new TF–target-gene interactions that are currently missing in the gold standard. Again, these dependencies would be regarded as false positives. This problem has been already discussed in the literature^2^ suggesting to use the false positive and true negative measures with caution for this reason.

**Figure 3:**
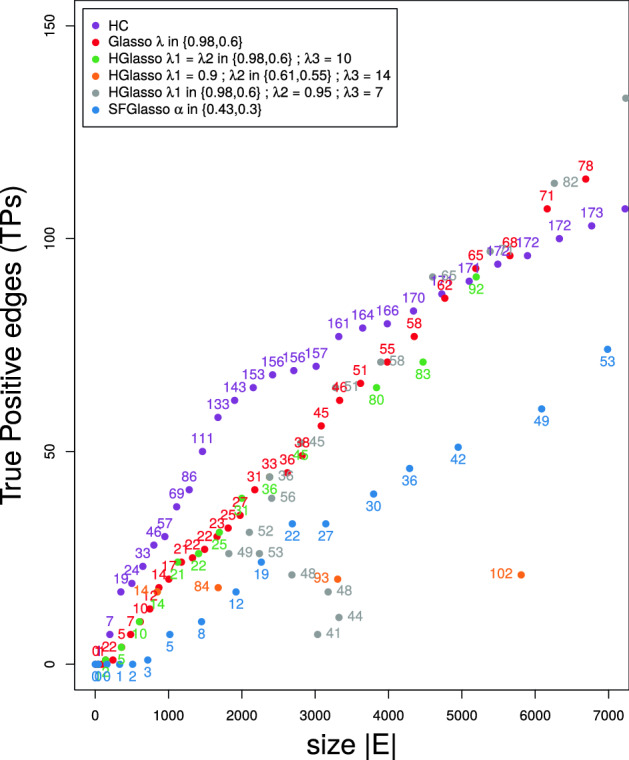
True positives (dots) and amount of transcription factors (numbers) w.r.t. the networksize $|\mathrm {E}|$ as encountered from the M3D microarray dataset by HC (purple), Glasso with $\lambda \in \{0.98,0.6\}$ (red), HGlasso with $\lambda _1 = \lambda _2 \in \{0.98,0.6\}$, $\lambda _3 = 10$ (green), HGlasso with $\lambda _1 = 0.9$, $\lambda _2 \in \{0.61,0.55\}, \lambda _3 = 14$ (orange), HGlasso with $\lambda _1 \in \{0.98,0.6\}$, $\lambda _2 = 0.95, \lambda _3 = 7$ (grey), and SFGlasso with $\alpha \in \{0.43, 0.3\}$ (blue).

Similarly to the previous analysis for the in silico dataset, Fig. 3 shows the results on TPs and TFs for the RegulonDB with respect to the number of total estimated edges $|\mathrm {E}|$ in the network for the four algorithms. Dots indicate the amount of TPs and the dot labels, in the form of numbers, indicate the amount of TFs found in the belonging network. Figure 3 shows that the UNMs obtained with HC contain the highest amount of TPs and TFs for sparse networks up to 5000 edges. The biggest gain of TPs with respect to the amount of edges $|\mathrm {E}|$ occurs in the range from 1000 to 1800 edges. In this range also the amount of recovered TFs is highest (around 20 new TFs per 100 added edges). The standard Glasso recovers less TP edges than HC in sparse networks. The recovering rate is especially low with respect to HC in the range of 1000–1800 edges and is probably related to the fact that in this range Glasso only recovers between 1 and 2 new TFs per 100 edges. Similarly to the case of the in silico dataset, the amount of TPs found with Glasso exceeds HC values for big networks (consisting of more than 5000 edges). In the discussion we consider in more detail the strategy of Glasso and HC to discover TPs.

In contrast to the in silico network, Glasso and variants discover relatively few TFs in sparse networks. Moreover, the Glasso variants do not all perform equally well with respect to the evidence of the RegulonDB network. The performance of HGlasso (and whether it can outperform Glasso or not) depends on the network size and the combination of the values of the parameters $\lambda _1,\lambda _2$, and $\lambda _3$. Take for example the combination $\lambda _1 = 0.9$, $\lambda _2$ = variable, and $\lambda _3 = 14$ (orange dots). These networks consist of more TFs than standard Glasso networks, but less TPs are found. The impact of the relaxed penalty term in the score function for hubs is thus visible, but not realized through the caption of more TPs by the HGlasso algorithm. The best results of HGlasso are obtained with $\lambda _1$ = variable, $\lambda _2 = 0.95$, and $\lambda _3 =7$ (grey dots); medium networks (up from 3000 edges) with these parameters not only consist of more TFs, but also outperform the standard Glasso on TPs. Finally, we see that SFGlasso performs worse than the Glasso and HGlasso on both the amount of TPs and TFs. The discovery rate of TFs is slow and, in case of real-world data, this results in few added TPs. It seems that the power-law degree distribution imposed by the SFGlasso algorithm does not fit well to the dependency structure determined by TFs in the M3D microarray data.

The difference in sample size (n = 805 for in silico and n = 466 for RegulonDB) and/or the difference in underlying dependency structure of the in silico and RegulonDB could have caused the differences in behaviour of Glasso algorithms on the two datasets. Note that, in the end, data is our principle resource for analysing new unknown gene regulatory networks. This type of resource brings forward the need for computational methods that can extract a generalizable model from (a single) realisation(s) and, at the same time, faithfully reveal the various emergent phenomena like co-expression, that are relevant in the system.

**Figure 4:**
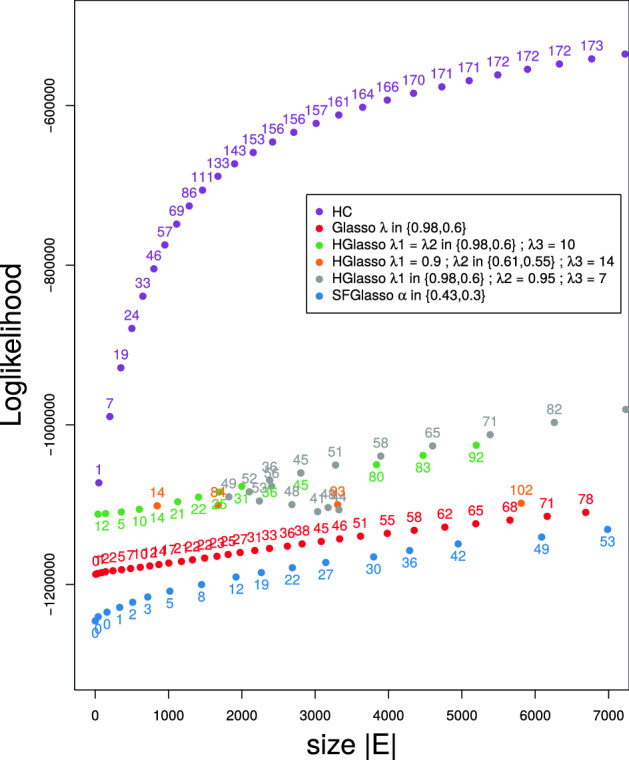
Log-likelihood values (dots) and amount of transcription factors (numbers) w.r.t. the networksize $|\mathrm {E}|$ as encountered from the M3D microarray dataset by HC (purple), Glasso with $\lambda \in \{0.98,0.6\}$ (red), HGlasso with $\lambda _1 = \lambda _2 \in \{0.98,0.6\}$, $\lambda _3 = 10$ (green), HGlasso with $\lambda _1 = 0.9$, $\lambda _2 \in \{0.61,0.55\}$, $\lambda _3 = 14$ (orange), HGlasso with $\lambda _1 \in \{0.98,0.6\}$, $\lambda _2 = 0.95$, $\lambda _3 = 7$ (grey), and SFGlasso with $\alpha \in \{0.43, 0.3\}$ (blue).

To chart networks on their data information content, in the next, we implement a simple probabilistic evaluation measure that appreciates faithfulness of algorithms to observational data sources. We measure the relative probabilistic accuracy of a single learned UNM with respect to another learned UNM by their difference in log-likelihood. The log-likelihood quantifies how well the model explains the data in terms of the likelihood of the available data given the particular probabilistic model (see “Methods” section “Log-likelihood definition and calculation”). Figure 4 displays the log-likelihood of the networks versus the amount of total links in the networks. In the case of Gaussian PNMs the number of parameters in the probabilistic model grows linearly in function of the amount of edges. Hence, the log-likelihood relative to the number of edges of Gaussian PNMs also provides information on models’ parsimony. For easy comparison with Fig. 3, we again placed the number of integrated TFs as dotlabels.

Figure 4 shows that the log-likelihood curve of HC rapidly improves until 2000 edges and establishes between 2000 and 7000 edges. Note that the same is true for the number of found TFs, growing rapidly until 2000 edges, reaching 153 TFs, and than slowing down until 7000 edges, reaching the total amount of 173 TFs. The true positives curve in Fig. 3 also begins to flatten around 2000 edges. Hence, in the case of HC, the validation measures TPs and TFs can be directly associated with the amount of information that is extracted from the microarray data and captured in the network.

In the case of H-, SF-, and Glasso the relation between log-likelihood and evaluation measures is more complex. The Glasso log-likelihood curve remains far apart from the HC curve for small and medium networks. From Fig. 3 we learn that Glasso values of evaluation measures finally approach HC values when including more and more edges. Figure 4 illustrates that this is not true (or in any case not true for regularized networks) for log-likelihood values. In the discussion we go into the relation between log-likelihood and TPs for Glasso and HC.

All HGlasso networks score better on log-likelihood values than the standard Glasso. For HGlasso networks that improve Glasso networks on TPs (e.g., HGlasso networks up from 3000 edges with $\lambda _2 = 0.95$ and $\lambda _3 = 10$; grey dots) this betterment is even more pronounced. Still, however, HC log-likelihood values are not reached neither at small nor at medium edge size by the adapted HGlasso algorithm. Similar to the case of TPs, SFGlasso scores worse than Glasso on log-likelihood values for small to medium networksizes. We do observe that the more edges are added to the SFGlasso network, the closer log-likelihood values become to Glasso values.

### Illustration of network structure

**Figure 5:**
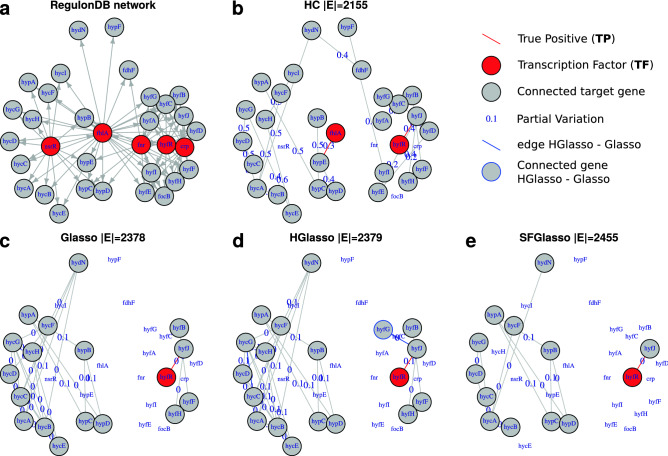
Reference and estimated networks. Panel (**a**) is the part of the reference RegulonDB network that contains the two TF encoding genes hyfR and fhlA and their direct neighbours. Panel (**b**–**e**) are the corresponding sub-networks of networks that are learned with respectively HC, (H)Glasso, and SFGlasso. In panel (**a**) TF encoding genes are coloured in red. In (**b**–**e**) TF encoding genes are coloured in red only when included in the network and connected genes are coloured grey. Red edges in (**b**–**e**) have a direct counterpart in panel (**a**) (i.e. red edges are TPs). In panel (**d**) blue edges and blue vertex closure indicate edges and vertex that are included in the HGlasso network but not in the Glasso network of the same size, whereas grey edges and black vertex closures indicate edges and vertices that exist in both networks.

To illustrate the above results on the performance of the methods, we zoom into a part of the reference RegulonDB network that contains the two TF encoding genes hyfR and fhlA—there is a similar performance in other portions of the network—and we compare with the networks learned from M3D with the four structure learning algorithms. Panel (a) in Fig. 5 shows the two TF encoding genes hyfR and fhlA (coloured in red) and their direct neighbours in the RegulonDB network (among the direct neighbours are three other TFs that are also colored in red: crp, fnr and nsrR).

The RegulonDB is a directed network and symmetric dependencies that could exist between children of hyfR and/or fhlA due to their common parent are not documented in the RegulonDB and hence are not displayed in panel (a). Panels (b–e) show the same subset of nodes as panel (a) and form part of networks that are learned from M3D by respectively HC, Glasso, HGlasso, and SFGlasso. These networks are all (converted to, in the case of HC) UNMs in which edges are undirected and represent direct dependency between two variables (the edges represent non zero entries in the symmetric precision matrix). The exact encoding of dependencies in the networks is described in the “Methods” section “Dependencies in PN”. The grey nodes indicate genes that are integrated in the network and red edges indicate edges that find their direct counterpart in the reference network in panel (a), i.e. edges that are denominated TPs. The edge-labels display the weight of the edges, the estimated partial variation. In panel (d) blue edges and blue vertex closure indicate edges and vertex that are included in the HGlasso network but not in the Glasso network in panel (c) of similar size, whereas grey edges and black vertex closures indicate edges and vertices that exist in both networks.

The results in the above subsections are illustrated in the subnetworks. In a sparse network, Hill Climbing (panel (b)) discovers both TF encoding genes, hyfR and fhlA and with them two TP edges between respectively hyfR and hyfJ and fhlA and hypE. Glasso, HGlasso, and SFGlasso (panels (c)–(e)) discover only one TF, hyfR, and find one TP related to hyfR.

Despite finding the most TPs, HC also discovers the most unique child–child dependencies between the target genes that are regulated by the transcription factors HyfR and FhlA. Child–child dependencies are not considered as TPs in the RegulonDB network, but do improve quality of UNM network structure. For example, the community $\{\mathrm {hyfA, hyfB}, \dots, \mathrm {hyfJ}\}$ and the TP between hyfR and hyfJ can be an indication that the set $\{\mathrm {hyfA}, \mathrm {hyfB}, \dots, \mathrm {hyfJ}\}\setminus \{\mathrm {hyfJ}\}$ is also progeny of HyfR.

Glasso finds less variety of child–child dependencies between nodes that are regulated by HyfR and FhlA. Instead, the sub groups of children are interconnected with more edges between them. These edges are probably true, but, from an information theoretic perspective, are redundant in the sense that they lower entropy of the community structure in the network (see also^28^). Moreover, the values of the parameters, the partial variation, in Glasso differ in magnitude with the values of the parameters in HC. The low partial variation as estimated by (H)Glasso lower log-likelihood values with respect to HC. In the discussion we come back to the estimation of the magnitude of the parameters and its impact on log-likelihood values.

In panel (d), HGlasso improves the network structure with respect to Glasso with an additional connected gene hyfG while using the same amount of edges. Also, parameter estimation seems more accurate as HGlasso includes higher partial variation values. In general, the inclusion of more significant edges (for some combinations of $\lambda _1,\lambda _2,\lambda _3$) and more precise parameter estimation alter the log-likelihood values of HGlasso with respect to Glasso, showed in Figs. 3 and  4.

The SFGlasso in panel (e) on the other hand excludes four more genes than the Glasso. Not many dependencies are found in this part of the sub network. Thereby, the estimation of parameters is worse in this part of the sub network. Partial variations are of even smaller magnitude with respect to Glasso. These results are in accordance with Fig. 3 showing that SFGlasso networks are less complete than (H)Glasso networks and with Fig. 4 showing that the least amount of information can be induced from SFGlasso networks.

## Discussion

Our results on sparse and medium-size networks showed high log-likelihood values for HC with respect to the standard Glasso and its modifications. Moreover, the results for sparse networks (up to 5000 edges) show that the HC algorithm finds most interactions for which evidence exists (in the form of TPs). We first discuss the log-likelihood and TP results in the light of the topology of the learned networks and the score functions that are integrated in the algorithms. Then, we analyze the results of HC in the framework of two additional partial-variation learning algorithms (SPACE and ESPACE) that are evaluated in the literature^30^ using the same *E. coli* dataset and an similar evaluation procedure. Finally, we discuss some generalities about GBNs that are learned with HC and applied on high dimensional real-world data^28^.

When analyzing log-likelihood values, we should first keep in mind that not all interactions that can be inferred from the M3D are equally informative and thus not contribute equally to the log-likelihood. It is intuitive that TF–target-gene interactions determine the backbone structure of the M3D data, whereas local interaction structure between children of the same TFs is less informative with respect to the complete M3D database. Therefore, the HC algorithm produces network topologies that contribute to high log-likelihood values, finding relatively more TPs and much more TFs than Glasso and its modifications in sparse networks, and just enough links to determine local interaction structure. Secondly, accurate estimation of the incorporated parameters (or the weight of the edges) alters log-likelihood values. The HC algorithm is able to accurately estimate the parameters, thanks to the $l_0$ penalty used in the score function that only penalizes the *amount* of parameters and not their values. The $l_1$ regularization in Glasso, on the other hand, while penalizing the amount of parameters, also penalizes their values and, thus, imposes a structural bias on the selected parameters in the Gaussian JPD function, resulting in inaccurate parameter estimation and substantially lower log-likelihood values. The score functions including $l_1$ or $l_0$ penalization are described in detail in “Methods” sections “Learning PN structure from data” and “Learning BN structure from data”.

The reasons for HC being more efficient than Glasso and modifications in sparse networks have to do with the integrated score functions and the steps in the learning algorithms. In every iteration, HC incorporates a parameter/edge maximizing the score function composed by the log-likelihood and the $l_0$ penalization terms. On the one hand, there are only a few high informative links (from TFs) that alter the log-likelihood substantively. HC is designed to optimize log-likelihood with the minimum amount of parameters and, thus, includes first TFs and informative local links that enlarge communities. On the other hand, the Glasso score function includes the log-likelihood part and the $l_1$ penalization term. Local links generally have a stronger direct correlation (see Fig. 5) and Glasso includes more of them, whether informative or uninformative, as local links ‘remain’ under application of the statistical criterion. More informative links between TFs and target genes have weaker direct correlation and in sparse networks Glasso shrinks those links to zero.

The differences in results between Glasso and modifications can be explained using similar arguments. Due to the adapted $l_1$ regularization, HGlasso performs better on log-likelihood values than the standard Glasso for every combination of initial parameters $\lambda _1,\lambda _2, \lambda _3$. The new score function alleviates the penalization on parameters that are related to hubs, resulting in higher log-likelihood values with respect to the standard Glasso. HGlasso, however, still depends heavily on $\lambda _1$ (the penalty term for non hubs) to introduce overall sparsity, and thus can not reduce the mean parameter bias sufficiently to reach HC values for small and medium networks. With respect to the measure of true positives, the performance of HGlasso (including whether it outperforms Glasso) depends on the combination of tuning parameters and on the network size. A successful combination of tuning parameters leads HGlasso to finding more TPs. These networks generally contain more TFs than the associated Glasso network. However, the incorporation of more TFs does not always lead to more true positives in the network. We found no standard method to discover combinations of initial parameters with which Glasso could be outperformed. Finding such a combination is a time consuming trial and error process.

The algorithm SFGlasso does incorporate a hub structure, but the networks generally contain less TFs than Glasso and the combinations of HGlasso. As resulting from the low log-likelihood values, the algorithm thus selects ‘wrong’ hubs and also places great bias on the estimates of informative links. These results may have to do with the poor adjustment of SFGlasso to high dimensional data. Generally, in scale-free networks, the coefficient $\alpha$ in the power-law with which the degree distribution decays lies between 2 and 3. However, we had to reduce $\alpha$ to values below 0.5 as otherwise the resulting networks resulted empty. For $\alpha \le 1$ the degree distribution is not generally well-defined and requires the existence of a maximum degree $k_{max}$ to be normalizable. In this work we only display results obtained with two iterations of the algorithm, because using more than two iterations the Glasso algorithm took extraordinary long time to converge. In this sense, one must be careful with the interpretation of the power-law resulting from SFGlasso.

The analysis of HGlasso and SFGlasso with respect to Glasso shows that the intrinsic complexity structure of the complex system is not (fully) represented by their ad hoc procedure to impose a hub structure and a power-law degree distribution in the network. Therefore, we can conclude that the GRN underlying *E. coli* is either not fully characterized by a power-law degree distribution and/or hub nodes or the algorithms fail to faithfully model these characteristics from high dimensional complex data.

Besides the standard Glasso and variants used in this paper, other advanced algorithms such as SPACE and ESPACE^30^ have been been applied on an older version of the microarray data. The algorithm SPACE uses sparse covariance selection techniques and was especially designed for the large-*p*-small-*n* data in the framework of GRNs and for powerful identification of hubs^34^. The ESPACE algorithm is an extension of the SPACE method and explicitly accounts for prior information on hub genes, which, in the case of *E. coli* data, yields knowing in advance that TFs are the highly connected nodes in the true *E. coli* GRN. We can loosely compare the HC algorithm with the performance of the algorithms SPACE and ESPACE. In particular, SPACE reports a precision of 4.35% in a network of 386 edges, and ESPACE reports a precision of 12.89% in a network of 349 edges^30^. In our work, HC shows a precision of 4.85% in a network of 350 edges. We may conclude with some precaution, as both M3D and the RegulonDB has been extended and updated meanwhile, that HC outperforms the exploratory algorithm SPACE, but can not compete with ESPACE that starts with prior information about the true hubs, i.e. information about the transcription factors.

Finally, the great power of HC when exploring *E. coli* data with unknown structure is that one can extract information of the true deterministic underlying network structure from every edge, as was illustrated at hand of the graphs in Fig. 5. The fact that no edge is redundant with respect to the dataset enables us to learn from every ‘false’ positive edge, whereas in the case of Glasso and variants this is not true: not every edge is data-significant neither tells us more about the network structure. The same conclusion was drawn for high dimensional climate data for which we found that HC provided an informative community structure that can be analyzed well with centrality measures (i.e. betweenness centrality, see^28^). The HC approach prioritizes an efficient edge distribution by favouring heterogeneous selection of edges between communities over uniform selection of edges that lie in communities. The resulting network topology allows for analysis with complex network measures; in the case of climate data these measures revealed spatially distant communities interconnected by teleconnections. Complex network centrality measures also have high potential applied on the PNMs learned with HC from real-world microarray data, especially when TF encoding genes are not known (i.e. no database similar to the RegulonDB exists); in this case to reveal candidate TFs and distinguish regulatory interactions from target-gene–target-gene interactions.

## Conclusions

The use of the Hill Climbing algorithm that arises in the context of Gaussian Bayesian networks offers a sound approach for the reconstruction of gene regulatory networks of high-dimensional-low-sample-size microarray data when no initial information is at hand about the underlying complex dependency structure. The HC algorithm picks only the most significant dependencies from the Gaussian data and in this manner naturally includes the complex dependency structure of the complex GRN that may consist of hubs, a scale-free degree distribution, outliers, a combination of the former or of other real-world characteristics. The algorithm naturally leaves out uninformative dependencies and variables.

The Bayesian network can easily be transformed to an undirected Gaussian probabilistic network model (i.e. a Gaussian Markov network or precision network), paying the price of some loss of information on the independence structure with respect to its initial directed Bayesian network. If one prefers an undirected PNM over a directed PNM—for easy interpretation due to symmetric links or for sparse estimation of the inverse covariance matrix—this study shows that the transformation from BN to PN is worth this loss of information for sparse networks as the PNs obtained by Hill Climbing outperform PNs obtained by state of the art PN-structure learning algorithms when applied to high-dimensional-low-sample-size (microarray) data that contains an unknown complex dependency structure.

This conclusion is drawn with respect to state-of-the-art PN structure algorithms that arise in the context of undirected Gaussian network models: the Glasso algorithm and variants of Glasso that are developed to integrate complex dependency structure. These algorithms model unnecessary dependencies at the expense of the probabilistic information in the network and of a structural bias in the probability function that can only be relieved including many parameters. In the case of the *E. coli* gene expression data used in this work, unnecessary dependencies also go at the expense of the amount of true positive edges, the last as judged by a reference network compounded of evidence gathered in the RegulonDB.

## Methods

### Gaussian probabilistic network models

The term refers to the choice of a multivariate Gaussian Joint Probability Density (JPD) function to associate graph edges with model parameters in a given PNM, such that the probabilistic model encodes in the JPD function a large number of random variables that interact in a complex way with each other by a graphical model. The multivariate Gaussian JPD function can take various representations in which dependencies between the variables are described by different types of parameters. The best known representation of the Gaussian JPD function is in terms of marginal dependencies, *i.e.* dependencies of the form $X_i,X_j|\emptyset$ as present in the covariance matrix ${\varvec{\Sigma }}$. Let ${\mathbf {X}}$ be a *N*-dimensional multivariate Gaussian variable then its probability density function ${\text {P}}({\mathbf {X}})$ is given by:

1 $$\begin{aligned} {\text {P}}({\mathbf {X}}) = (2\pi )^{-N/2}\det ({\varvec{\Sigma }})^{-1/2}\exp \{-1/2({\mathbf {X}}-{\varvec{\mu }})^\top {\varvec{\Sigma }}^{-1}({\mathbf {X}}-{\varvec{\mu }})\}, \end{aligned}$$

where ${\varvec{\mu }}$ is the *N*-dimensional mean vector and ${\varvec{\Sigma }}$ the $N \times N$ covariance matrix. In the following we describe in some detail two types of Gaussian PNMs, in which parameters reflect respectively direct dependencies $X_i,X_j|{\mathbf {X}}\backslash \{X_i,X_j\}$ and general conditional dependencies $X_i|{\mathscr {S}}$ with ${{\mathscr {S}}}\subseteq {\mathbf {X}}$ (direct dependencies are the least restrictive case of conditional dependencies).

### Probabilistic PN models

The Gaussian JPD function in Eq. (1) can be formulated more generally using a set of factors ${\varvec{\Phi }} = \{\phi _1({\mathscr {S}}_1),\dots ,\phi _k({\mathscr {S}}_k)\}$ that describe dependencies between arbitrary (overlapping) subsets of variables ${\mathscr {S}}_1,\dots ,{\mathscr {S}}_k$ which comply with $\cup _k {\mathscr {S}}_k = {\mathbf {X}}$. This representation of the JPD is called the Gibbs function and written as^31^

2 $$\begin{aligned} {\text {P}}(X_1,\dots ,X_N) = \frac{1}{Z}{\tilde{{\text {P}}}}(X_1,\dots ,X_N), \end{aligned}$$

with

3 $$\begin{aligned} {\tilde{{\text {P}}}}(X_1,\dots ,X_N) = \prod _{i=1}^{k} \phi _i({\mathscr {S}}_i) \qquad \text {and} \qquad Z = \sum _{i=1}^{N}{\tilde{{\text {P}}}}(X_1,\dots ,X_N). \end{aligned}$$

The Gibbs distribution where all of the factors are over subsets of single variables or pairs of variables is called a pairwise Markov network. The factors in a pairwise Markov network correspond to direct dependencies, i.e., dependencies of the form $X_i,X_j|{\mathbf {X}}\backslash \{X_i,X_j\}$. In a Gaussian distribution these dependencies are present in the inverse covariance matrix or precision matrix ${\varvec{\Sigma }}^{-1}$. The information form of the Gaussian JPD function in terms of the precision matrix ${\varvec{\Theta }} = {\varvec{\Sigma }}^{-1}$,

4 $$\begin{aligned} {\text {P}}({\mathbf {X}}, \mu = 0, {\varvec{\Theta }}) = (2\pi )^{-N/2}|{\varvec{\Theta }}|^{1/2}\exp \{-1/2\sum _{i}\theta _{ii}X_i^2-\sum _{i<j}\theta _{ij}X_iX_j\}, \end{aligned}$$

is equivalent to the Gibbs function in Eq. (2) with factors defined on every variable and every pair of variables, i.e. ${\varvec{\Phi }} = \{{\varvec{\Phi }}^n,{\varvec{\Phi }}^e\}$ with $\phi ^n_i = \exp \{-\frac{1}{2} \theta _{ii}X_i^2\}$ and $\phi ^e_{ij} = \exp \{\theta _{ij}X_i X_j\}$.

The corresponding Gaussian PNM in which the notion of variables and pairs of variables is extended to the notion of nodes and undirected edges in a graph is called the probabilistic PN model. The graph of a PN encodes the probability function in Eq. (4) as follows. Each node corresponds to a variable $X_i \in {\mathbf {X}}$, the presence of an edge $X_i - X_j$ implies the presence of the factor $\phi ^e_{ij}$ in ${\text {P}}({\mathbf {X}})$ and direct dependency of $X_i$ and $X_j$. Moreover, the absence of an arc between $X_i$ and $X_j$ in the graph implies the absence of the factor $\phi ^e_{ij}$ in ${\text {P}}({\mathbf {X}})$ and, thus, the existence of a set of variables ${{\mathscr {S}}} \subseteq {\mathbf {X}}\backslash \{X_i,X_j\}$ that makes $X_i$ and $X_j$ conditionally independent in probability^31^.

The graph structure of PNs in this work is estimated simultaneously with the values of the parameters in ${\varvec{\Theta }}$ that define this structure. This simultaneous learning process is explained in “Methods” section “Learning PN structure from data”.

### Probabilistic BN models

Alternatively, the ${\text {P}}({\mathbf {X}})$ in Eq. (1) can be characterized with conditional dependencies of the form $X_i|{\mathscr {S}}$ with ${{\mathscr {S}}}\subseteq {\mathbf {X}}$. The representation of the JPD is then a product of conditional probability densities:

5 $$\begin{aligned} {\text {P}}(X_1,\dots ,X_N) = \prod _{i=1}^{N} {\text {P}}_i(X_i {\text {|}}\Pi _{X_i}) \end{aligned}$$

with

6 $$\begin{aligned} {\text {P}}(X_i {\text {|}}\Pi _{X_i}) \sim \mathcal{N}\left( \mu _i + \sum _{j|X_j \in \Pi _{X_i}}\beta _{ij}(X_j-\mu _j), \; \tfrac{1}{\nu _i}\right) \end{aligned}$$

whenever the set of random variables $\{X_i {\text {|}}\Pi _{X_i}\}_{i\in N}$ is independent^35^. In this representation $\mathcal{N}$ is the normal distribution, $\mu _i$ is the unconditional mean of $X_i$, $\nu _i$ is the conditional variance of $X_i$ given the set $\Pi _{X_i}$ and $\beta _{ij}$ is the regression coefficient of $X_j$, when $X_i$ is regressed on $\Pi _{X_i}$. We call $\Pi _{X_i}$ the parentset of variable $X_i$.

The corresponding Gaussian PNM in this case is the probabilistic BN model. The graph of a BN model is a $\mathrm {DAG}$ encoding the corresponding probability distribution as in Eq. (5). Each node corresponds to a variable $X_i \in {\mathbf {X}}$, the presence of an arc $X_j \rightarrow X_i$ implies the presence of the factor ${\text {P}}_i(X_i|\dots X_j \dots )$ in ${\text {P}}({\mathbf {X}})$, and thus conditional dependence of $X_i$ and $X_j$. Moreover, the absence of an arc between $X_i$ and $X_j$ in the graph implies the absence of the factors ${\text {P}}_i(X_i|\dots X_j \dots )$ and ${\text {P}}_j(X_j|\dots X_i \dots )$ in ${\text {P}}({\mathbf {X}})$ and, thus, the existence of a set of variables ${{\mathscr {S}}} \subseteq {\mathbf {X}}\backslash \{X_i,X_j\}$ that makes $X_i$ and $X_j$ conditionally independent in probability^31,36^.

The graph structure of the BN identifies the parentset $\Pi _{X_i}$ in Eq. (5). With this structure available, one easily learns the corresponding parameter set $({\varvec{\beta }},{\varvec{\nu }})$; in our case parameters $\beta _{ij}$ and $\nu _i$ are a maximum likelihood fit of the linear regression of $X_i$ on its parentset $\Pi _{X_i}$. To estimate parameter values from graph structure we use the appropriate function in the R-package bnlearn^37^. The challenge of learning the graph structure is explained in “Methods” section “Learning BN structure from data”.

### Learning PN structure from data

A Precision Network (PN) is learned with the help of a structure learning algorithm that estimates the inverse covariance matrix, i.e. the precision matrix ${\varvec{\Sigma }}^{-1}$ of the underlying Gaussian distribution. Converted into binary format, the estimate ${\varvec{\Theta }}$ of ${\varvec{\Sigma }}^{-1}$ provides the undirected adjacency matrix ${\varvec{A}}$ of a pairwise Markov Network. From the adjacency matrix ${\varvec{A}}$ of the graph of a PN the structure of the factor-set ${\varvec{\Phi }} = \{{\varvec{\Phi }}^n,{\varvec{\Phi }}^e\}$ of the associated Gaussian JPD function (outlined in Eq. (4)) can be directly read off. In the “Methods” section “Probabilistic PN models” is explained how pairwise Markov networks encode the corresponding Gaussian PNM.

The Graphical lasso (Glasso) can be regarded as the default structure learning algorithm learning PNs for large-*p*-small-*n* datasets. Glasso is a score-based algorithm based on a convex score. This score is basically made up by the maximum likelihood estimate of the precision matrix of a Gaussian probability function to which an $l_1$ penalty term is added^15^:

7 $$\begin{aligned} Score ({\varvec{\Theta }}, {\mathbf {S}},\lambda ) = \log (\det {{\mathbf {S}}{\varvec{\Theta }}}) - tr({\mathbf {S}}{\varvec{\Theta }}) - \lambda \Vert {\varvec{\Theta }}\Vert _1. \end{aligned}$$

Here ${\varvec{\Theta }} = {\varvec{\Sigma }}^{-1}$, ${\mathbf {S}}$ is the sample covariance matrix calculated directly from the data ${\mathscr {D}}$ and $\lambda$ a scalar, the penalization coefficient. Networks of different sizes can be generated by varying the penalization parameter $\lambda$. In this work we generate networks with zero edges to complete networks by varying $\lambda$ from 1 to 0. A short outline of the steps in the Graphical lasso algorithm is given in “Methods” section “Learning with hill climbing and glasso”. The Glasso function is implemented in the R-package glasso^15^.

The Hub Graphical lasso (HGlasso) learns a PN that consist of hub nodes combining a lasso ($l_1$) penalty and a sparse group lasso ($l_2$) penalty^38^. The estimated inverse covariance matrix ${\varvec{\Theta }}$ can be decomposed as ${\varvec{\Theta }} = {\mathbf {Z}} + {\mathbf {V}} + t({\mathbf {V}})$, where ${\mathbf {Z}}$ is a sparse matrix and ${\mathbf {V}}$ is a matrix that contains hub nodes. The belonging score is

8 $$\begin{aligned} Score ({\varvec{\Theta }}, {\mathbf {S}},\lambda _1,\lambda _2,\lambda _3)&= {} \log (\det {{\mathbf {S}}{\varvec{\Theta }}}) - tr({\mathbf {S}}{\varvec{\Theta }}) \nonumber \\&- \lambda _1 \Vert {\mathbf {Z}}\Vert _1 - \lambda _2\Vert {\mathbf {V}} - \text {diag}({\mathbf {V}})\Vert _1 \nonumber \\&-\lambda _3\sum _{j = 1}^N \Vert ({\mathbf {V}} - \text {diag}({\mathbf {V}}))_j\Vert _2, \end{aligned}$$

with ${\varvec{\Theta }}$ restricted to ${\varvec{\Theta }} ={\mathbf {V}}+{\mathbf {V}}^T+{\mathbf {Z}}$. In this score $\lambda _3$ controls the selection of hub nodes, and $\lambda _2$ controls the sparsity of each hub nodes’ connections to other nodes. We obtain networks of different sizes by varying $\lambda _1,\lambda _2$, and $\lambda _3$. The HGlasso function is implemented in the R-package hglasso^38^.

The Scale-Free Graphical lasso (SFGlasso) aims to include even more structural information than mere sparsity or hubs^26^. Hubs are expected in this type of network but the focus lies on learning models that possess the so-called scale-free property; a property often claimed to appear in real-world networks. This feature is mathematically expressed by a degree distribution *p*(*d*) that follows a powerlaw: $p(d)\propto d^{-\alpha }$ (up from a certain degree *d*). In the score function of the standard Glasso, the $l_1$ edge regularization is replaced with a power law regularization. The objective score function is:

9 $$\begin{aligned} Score ({\varvec{\Theta }}, {\mathbf {S}},\alpha ,\beta )= & {} \log (\det {{\mathbf {S}}{\varvec{\Theta }}}) - tr({\mathbf {S}}{\varvec{\Theta }}) \nonumber \\&-\alpha \sum _i\log (\Vert {\varvec{\Theta }}\lnot i\Vert _1 +\varepsilon _i) - \beta \sum _i|\theta _{ii}|, \end{aligned}$$

with ${\varvec{\Theta }}_{\lnot i} = \{\theta _{ij}|j\ne i\}$. This score function is not convex, a requirement to use Glasso, however can be proven to be monotone increasing. The score $Score ({\varvec{\Theta }}, {\mathbf {S}},\alpha ,\beta )$ is sequentially improved by elements of the sequence ${\varvec{\Theta }}^{n}$ that iteratively maximize the following re-weighted convex $l_1$ regularization problems:

10 $$\begin{aligned} Score ({\varvec{\Theta }}^{n+1},{\mathbf {S}},\lambda _{ij})= & {} \log (\det {{\mathbf {S}}{\varvec{\Theta }}^{n+1}}) - tr({\mathbf {S}}{\varvec{\Theta }}^{n+1})\nonumber \\&- \sum _{i\ne j}\lambda _{ij}|\theta ^n_{ij}|-\beta \sum _i|\theta ^n_{ii}|, \end{aligned}$$

where $\lambda _{ij} = \alpha (\frac{1}{\Vert {\varvec{\Theta }}^n_{\lnot i}\Vert _1+\varepsilon _i}+\frac{1}{\Vert {\varvec{\Theta }}^n_{\lnot j}\Vert _1 + \varepsilon _j}).$ This re-weighting reduces regularization coefficients of nodes with high degree, encouraging the appearance of hubs with high degree.

Following the set up in the experiment section of Ref.^26^ we take $\beta _i = 2\alpha /\varepsilon _i$ and $\varepsilon _i$ equal to $\theta _{ii}$ estimated in the last iteration, in this way $\varepsilon _i$ is of the same magnitude of $\Vert {\varvec{\Theta }}^n_{\lnot j}\Vert _1$. To optimize Eq. (10) and find ${\varvec{\Theta }}^{n+1}$ we iteratively use the glasso function in the R-package glasso with $\lambda = \lambda ({\varvec{\Theta }}^{n})$, defined above.

### Learning BN structure from data

The graph of a BN is estimated with the help of a structure learning algorithm that finds the conditional dependencies between the variables and encodes this information in a DAG. Graphical (dis)connection in the DAG implies conditional (in)dependence in probability (see “Methods” section “Dependencies in BN”). From the structure of a BN a factorization of the underlying JPD function ${\text {P}}({\mathbf {X}})$ of the multivariate random variable ${\mathbf {X}}$ (as given by Eq. (5)) can be deduced. In the “Methods” section “Probabilistic BN models” is explained how networks can be extended to their corresponding probabilistic network model.

In general there are three types of structure learning algorithms: constraint-based, score-based, and hybrid structure learning algorithms—the latter being a combination of the first two algorithms.

Constraint-based algorithms use conditional independence tests of the form $Test (X_i,X_j|{\mathscr {S}};{\mathscr {D}})$ with increasingly large candidate separating sets ${\mathscr {S}}_{X_i,X_j}$ to decide whether two variables $X_i$ and $X_j$ are conditionally independent. All constraint-based algorithms are based on the work of Pearl on causal graphical models^39^ and its first practical implementation was found in the Principal Components algorithm^40^. In contrast, score-based algorithms apply general machine learning optimization techniques to learn the structure of a BN. Each candidate network is assigned a network score reflecting its goodness of fit, which the algorithm then attempts to maximise^41^. In Ref.^29^ we compared structure learning algorithms belonging to the three different classes on accuracy and speed for high dimensional complex data. We found that score-based algorithms perform best. Algorithms in this class are able to handle high-variable-low-sample-size data and find networks of all desired sizes. Constraint-based algorithms can only model complex data up to a certain size and, as a consequence, for climate data they only reveal local network topology. Hybrid algorithms perform better than constraint-based algorithms on complex data, but worse than score-based algorithms.

In this work we use a simple score-based algorithm, the Hill Climbing (HC) algorithm^41^, to learn BN structure. The HC algorithm starts with an empty graph and iteratively adds, removes or reverses an edge maximizing the score function. This algorithm is formalized in “Methods” section “Learning with hill climbing and glasso”. HC is implemented in the R-package bnlearn.

We used the Bayesian Information Criteria (BIC) (corresponding to $\mathrm {BIC}_0$ in Ref.^29^) score, which is defined as:

11 $$\begin{aligned} \mathrm {BIC}({\mathscr {G}}; {\mathscr {D}}) = \sum _{i=1}^N \left[ \; \log {\text {P}}(X_i {\text {|}}\Pi _{X_i}) - \frac{|{\varvec{\Theta }}_{X_i}|}{2}\log n \;\right] , \end{aligned}$$

where ${\mathscr {G}}$ refers to the graph (DAG) for which the BIC score is calculated, P refers to the probability density function that can be deduced from the graph (see “Methods” section “Probabilistic BN models”), $\Pi _{X_{i}}$ refer to the parents of $X_i$ in the graph (*i.e.* nodes Y with relation $Y \rightarrow X_i$ in the graph) and $|{\varvec{\Theta }}_{X_{i}}|$ is the amount of parameters of the local density function ${\text {P}}(X_i {\text {|}}\Pi _{X_i})$.

### Dependencies in BN and PN structure

**Figure 6:**
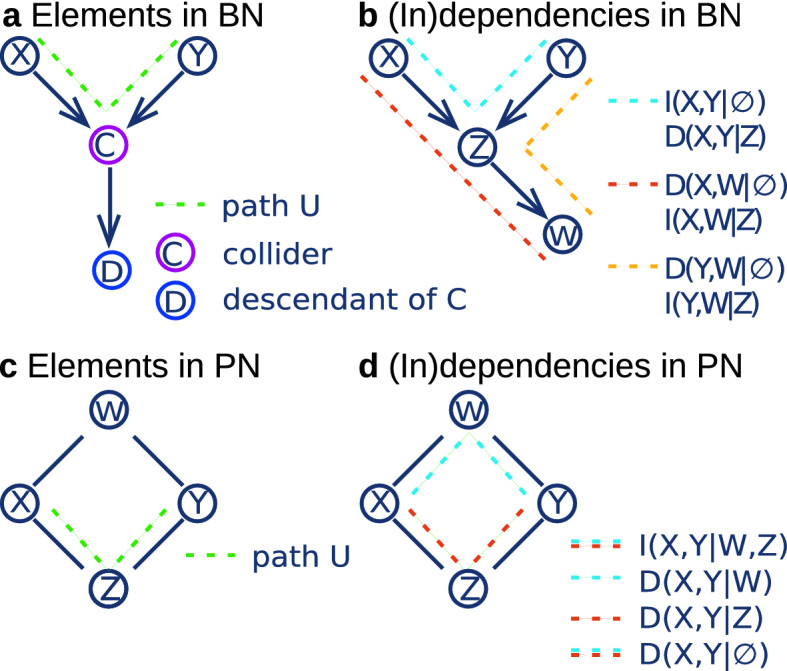
(**a**) and (**c**): Nomenclature of elements in respectively a Bayesian Network (BN) and a Precision Network (PN). (**b**) and (**d**): Some (in)dependencies in simple BN and PN consisting of four nodes *X*, *Y*, *Z* and *W*. In (**b**) two sets of nodes are dependent given a third if conditions (1) and (2) in the main text are fulfilled. On the one hand, the conditional relationship *X*, *Y*|*Z* and the marginal relationships $X,W|\emptyset$ and $Y,W|\emptyset$ satisfy conditions (1) and (2), so that we have $\mathrm {D}(X,Y|Z)$, $\mathrm {D}(X,W|\emptyset )$, and $\mathrm {D}(Y,W|\emptyset )$. On the other hand, the marginal relationship $X,Y|\emptyset$ violates condition (1) and the conditional relationships *X*, *W*|*Z* and *Y*, *W*|*Z* violate condition (2), so that we have $\mathrm {I}(X,Y|\emptyset )$, and $\mathrm {I}(X,W|Z)$ and $\mathrm {I}(Y,W|Z)$. In (**d**) the conditional relationships *X*, *Y*|*W*, *X*, *Y*|*Z*, and $X,Y|\emptyset$ satisfy the condition of graphical dependence in a PN, and hence the statements $\mathrm {D}(X,Y|W)$, $\mathrm {D}(X,Y|Z)$, and $\mathrm {D}(X,Y|\emptyset )$ hold. On the other hand the conditional relation *X*, *Y*|*W*, *Z* does not satisfy the condition of graphical dependence; there does not exist a path $\mathrm {U}$, such that neither *W* nor *Z* is not on *U*. Thus $\mathrm {I}(X,Y|W,Z)$ holds. In the following we give a formal proof of $\mathrm {D}(X,Y|Z)$ in (**b**): The conditioning set ${\mathscr {S}}$ exists of *Z*. The only path between *X* and *Y* is the blue path. Hence we declare the blue path U. *Z* is a collider and *Z* is in ${\mathscr {S}}$. There are no other colliders on U. Hence condition (1) is satisfied. *Z* is the only variable on U. And *Z* is a collider. Thus, U does not contain non-colliders. Hence condition (2) is satisfied. As condition (1) and (2) are satisfied we have that *X* and *Y* are dependent given *Z*, i.e. $\mathrm {D}(X,Y|Z)$.

In the following we describe how a BN (or DAG) and a PN (or pairwise Markov network) encode conditional dependencies. New nomenclature is indicated with an asterisk and illustrated in Fig. 6a–c.

### Dependencies in BN

In a BN two nodes *X* and *Y* are conditionally dependent given a set ${\mathscr {S}}$ (denoted by $\mathrm {D}(X,Y|{\mathscr {S}})$) if and only if they are graphically connected, that is, if and only if there exists a path $\text {U}^*$ between *X* and *Y* satisfying the following two conditions:

- *Condition (1)*: for every collider$^*$ C (node C such that the part of U that goes over C has the form of a V-structure, *i.e.* $\rightarrow C \leftarrow$) on U, either C or a descendant$^*$ of C is in ${\mathscr {S}}$.
- *Condition (2)*: no non-collider on U is in ${\mathscr {S}}$.

If the above conditions do not hold we call *X* and *Y* conditionally independent given the set ${\mathscr {S}}$ (denoted by $\mathrm {I}(X,Y|{\mathscr {S}})$). Marginal dependency between two nodes can be encoded by any path U with no V-structures. In Fig. 6b six conditional (in)dependence statements are highlighted in a simple DAG. In the caption of Fig. 6 one of the statements is proved at the hand of conditions (1) and (2).

### Dependencies in PN

In a PN two nodes *X* and *Y* are conditionally dependent given a set ${\mathscr {S}}$ (denoted by $\mathrm {D}(X,Y|{\mathscr {S}})$) if and only if there exists a path $\text {U}^*$ between *X* and *Y* satisfying: No node $Z \in {\mathscr {S}}$ is on $\text {U}$. If the above condition do not hold we call *X* and *Y* conditionally independent given the set ${\mathscr {S}}$ (denoted by $\mathrm {I}(X,Y|{\mathscr {S}})$). Marginal dependency between two nodes can be encoded by any path U. In Fig. 6d four conditional (in)dependence statements are highlighted in a simple pairwise Markov network.

### Learning with hill climbing and glasso


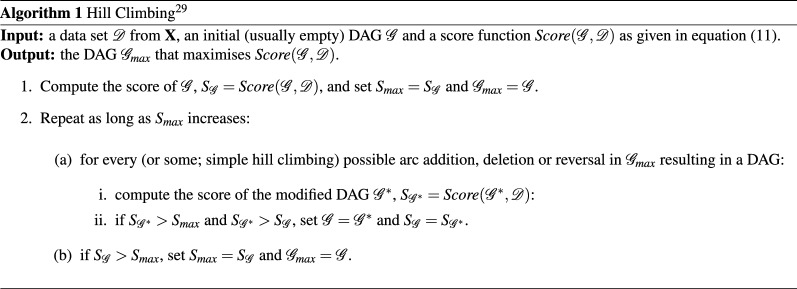
 
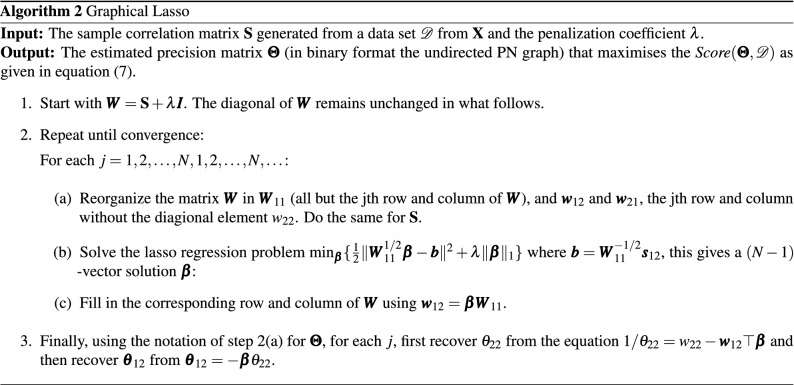


At the hand of Algorithms 1 and 2 we outline Hill Climbing and Graphical Lasso. For a more detailed description—and explanation of the equalities in Glasso—we respectively refer the reader to Refs.^15,41^. The input of both algorithms consists of the dataset $\mathcal D$ (the sample correlation matrix ${\mathbf {S}}$ in Algorithm 2 is just $(1/(n-1)){\mathcal{D}^\top } \mathcal D$ for standardized variables) consisting of *n* independent samples of the multi Gaussian variable ${\mathbf {X}}$ and a score function to optimize. The output of HC is a DAG, whereas the output of Glasso is the estimated precision matrix ${\varvec{\Theta }}$, which, in binary format, is the adjacency matrix of the associated undirected graph.

Hill Climbing simply visits all (or some; ‘simple’ Hill Climbing) neighbouring networks that differ on one edge of the current network (step 1) and moves then to the network with highest score—or directly to the first network found with better score in the case of simple HC (step 2). The algorithm stops when no neighbouring network has higher score than the current network (this could be at a local optimum). The optimized implementation of HC in bnlearn that was used in this work uses score caching, score decomposability and score equivalence to reduce the number of duplicated tests. When datasets of higher dimensionality are addressed, other efficient versions of HC can be considered^42^.

Glasso transforms the initial score function (Eq. 7) to a lasso problem and applies a coordinate descent approach to solve the problem: the algorithm fixes all dependencies in the current estimate of the correlation matrix ${\varvec{W}}$ except those of one variable (coordinate), i.e. except one column and row (step 2a). Then it estimates the dependencies of this variable that best solves the element wise lasso regression problem (step 2b) and fills in the corresponding row and column in the updated correlation matrix ${\varvec{W}}$ (step 2c). Next, it moves to the next coordinate and solves the same problem, this time with the former solution integrated in the fixed coordinates (integrated in ${\varvec{W}}_{11}$). This process (step 2) is repeated until convergence. Finally, in the last cycle, the row ${\varvec{\theta }}_{12}$ and diagonal element $\theta _{22}$ in ${\varvec{\Theta }}$ are recovered from ${\varvec{W}}$ and ${\varvec{\beta }}$ (step 3).

### Transformation of probabilistic BN model to probabilistic PN model

The purple arrow in Fig. 1 indicates that BNs can be transformed into PNs. The transformation of the DAG into an undirected graph is done applying *moralization*, i.e. including an undirected edge between any two nodes with a common child, and subsequently dropping edge directions. Thus, each set of parents and children $\{X_i,\Pi _i\}$ is a fully connected subset in the moral graph. The mapping may not be perfect and the dependencies of the resulting undirected moral graph may typically be an I-map of the original DAG (i.e. the moral graph is a minimum superset of the dependencies of the DAG meaning that there is no other undirected graph with less edges including all DAG dependencies but also that the inclusion of extra links and parameters in the transformation process slightly distorts the *in*-dependence structure of the network).

The moral graph of a BN can be asociated with more than one undirected PNM. To associate the moral graph with the special case of a probabilistic PN model that encodes the JPD formulated in Eq. (4), i.e. a *pairwise* Markov network, the parameterset of the initial BN has to be transformed in order to obtain the precision matrix. The following equality between the precision matrix ${\varvec{\Theta }}$ and the parameters $({\varvec{\beta }},{\varvec{\nu }})$ of a Gaussian Bayesian Network holds^43^:

12 $$\begin{aligned} {\varvec{\Theta }} = {\varvec{\Theta }}({\varvec{\beta }},{\varvec{\nu }}) = ({\varvec{I}}-{\varvec{B}}){\varvec{\nu }}^{-1}({\varvec{I}}-{\varvec{B}})^\top . \end{aligned}$$

The new weights of the edges and parameters of the *pairwise* Markov Network are the entries of the precision matrix:

13 $$\begin{aligned} \theta _{ij} = \theta _{ji} = -\frac{\beta _{ij}(1-\beta _{jj})}{\nu _{j}}-\frac{\beta _{ji}(1-\beta _{ii})}{\nu _{i}} + \sum _{k \ne i,j}\frac{\beta _{ik}\beta _{jk}}{\nu _k} \end{aligned}.$$

The entry $\theta _{ij}$ is zero if there is no edge i – j in the moral graph. Occasionally, $\theta _{ij}$ can take the value of zero as a result of the matrix summation at the right hand side of Eq. (13).

In this work we moralize and obtain the precision matrix of all BNs that were learned with the Hill Climbing algorithm. In practice we use the R-packages bnlearn for the process of moralization and sparseBNutils^44^ for the extraction of the precision matrix.

### Evaluation measures

Following^2^, we define the recall of a PNM with edge size $|\mathrm {E}|$ as

$$\begin{aligned} \mathrm {recall} = \frac{\mathrm {TP}}{\mathrm {TP + FN}} = \frac{\mathrm {TP}}{P} \end{aligned},$$

where $\mathrm {TP}$ is the number of true positives in the PNM, FN the number of false negatives in the PNM and *P* is the number of positives/edges in the directed RegulonDB network or the in silico network, i.e. $P = 3387$ and $P = 4012$, respectively. Moreover, we define the precision of a PNM with edge size $|\mathrm {E}|$ as

$$\begin{aligned} \mathrm {precision} = \frac{\mathrm {TP}}{\mathrm {TP} + \mathrm {FP}} = \mathrm {\frac{TP}{|E|}} \end{aligned},$$

where FP is the number of False Positives in the network.

### Log-likelihood definition and calculation

The likelihood of the data ${\mathscr {D}}$, given a model ${\mathscr {M}}$ is the density of the data under the given model ${\mathscr {M}}$: ${\text {P}}({\mathscr {D}}{\text {|}}{\mathscr {M}})$. For discrete density functions the likelihood of the data equals the probability of the data under the model. The likelihood is almost always simplified by taking the natural logarithm; continuous likelihood values are typically small and differentiation of the likelihood function (with the purpose of a maximum likelihood search) is often hard. Log-likelihood values can be interpreted equally when the expression is used for model comparison and maximum likelihood search as the natural logarithm is a monotonically increasing function.

In the following we explain the calculation of the log-likelihood ${\mathscr {L}}({\mathscr {D}}|{\mathscr {M}}) = \log {\text {P}}({\mathscr {D}}|{\mathscr {M}})$ for a PNM (${\mathscr {M}}= \mathrm {PNM}$) for a dataset ${\mathscr {D}}$ formed by *n* independent data realizations ${\mathscr {D}}_k$, $k \in \{1, \dots , n\}$, of the *N*-dimensional random vector ${\mathbf {X}}$, with ${\mathscr {D}}_k = \{d^k_1\dots d^k_N\}$ and $d^k_i$ the *k*-th realization of variable $X_i \in {\mathbf {X}}$. We have

14 $$\begin{aligned} \log {\text {P}}({\mathscr {D}}{\text {|}}\mathrm {PNM})= & {} \log {\text {P}}({\mathscr {D}}_1, \dots , {\mathscr {D}}_n{\text {|}}\mathrm {PNM}) = \log \prod _{k=1}^{n}{\text {P}}({\mathscr {D}}_k{\text {|}}\mathrm {PNM}) \nonumber \\= & {} \sum _{k=1}^{n}\log {\text {P}}({\mathscr {D}}_k{\text {|}}\mathrm {PNM}) = \sum _{k=1}^{n}\log {\text {P}}_{\mathrm {PNM}}({\mathscr {D}}_k) \end{aligned}$$

with ${\text {P}}_{\mathrm {PNM}}$ the probability density function as modelled by the corresponding PNM with a Gaussian multivariate probability. In this work we consider two types of PNMs, precision and Bayesian PNMs, deduced from PNs and BNs graphs, respectively. In the case of a Gaussian PNM given by a PN we get:

15 $$\begin{aligned} {\mathscr {L}}_\mathrm {PN}({\mathscr {D}}{\text {|}}\mathrm {PNM}_\mathrm {PN})= & {} \sum _{k=1}^{n}\log {\text {P}}({\mathscr {D}}_k{\text {|}}\mathrm {PNM}_\mathrm {PN}) \nonumber \\= & {} \sum _{k=1}^{n}\log \{(2\pi )^{-N/2} \det ({\varvec{\Theta }})^{1/2} \nonumber \\&\times \exp \{-1/2\sum _{i}^{N}\theta _{ii}(d^k_i)^2-\sum _{i<j}\theta _{ij}d^k_id^k_j\}\}. \end{aligned}$$

Entries in the sum are evaluations of the multivariate normal density function and executed with the R-package mvtnorm^45^.

In the case of a Gaussian PNM given by a BN, from Eq. (5), we have

16 $$\begin{aligned} {\mathscr {L}}_{\mathrm {BN}}({\mathscr {D}}{\text {|}}\mathrm {PNM}_{\mathrm {BN}})= & {} \sum _{k=1}^{n}\log {\text {P}}({\mathscr {D}}_k{\text {|}}\mathrm {PNM}_{\mathrm {BN}}) \nonumber \\= & {} \sum _{k=1}^{n}\log \prod _{i=1}^{N} {\text {P}}_i(X_i = d^k_i {\text {|}}\Pi _{X_i} = d^k_{\Pi _{X_i}}) \nonumber \\= & {} \sum _{k=1}^{n}\sum _{i=1}^{N} \log {\text {P}}_i(X_i = d^k_i {\text {|}}\Pi _{X_i} = d^k_{\Pi _{X_i}}), \end{aligned}$$

where $d^k_{\Pi _{X_i}}$ is a subset of ${\mathscr {D}}_k$ containing the *k*-th data realization of the parentset $\Pi _{X_i}$ of $X_i$. From Eq. (6) we know that the conditional univariate densities in the sum in Eq. (16) are univariate normal and we execute them with the basic R-package stats.

## Data Availability

The in silico network and the simulated gene expression dataset analysed during the current study are available through the DREAM5 paper^2^ in Supplementary data 1. The microarray dataset ‘E_coli_v4_Build_6’ and the TF-gene interaction network dataset ‘network_tf_gene’ are available in the Many Microbe Microarray databases^1^, http://m3d.mssm.edu/norm/E_coli_v4_Build_6.tar.gz and in the Regulon Data Base^33^, http://regulondb.ccg.unam.mx/menu/download/datasets/files/network_tf_gene.txt, respectively.
